# Application of Parallel Factor Analysis (PARAFAC) to electrophysiological data

**DOI:** 10.3389/fninf.2014.00084

**Published:** 2015-01-30

**Authors:** S. Katharina Schmitz, Philipp P. Hasselbach, Boris Ebisch, Anja Klein, Gordon Pipa, Ralf A. W. Galuske

**Affiliations:** ^1^Systems Neurophysiology, Department of Biology, Technische Universität DarmstadtDarmstadt, Germany; ^2^Department of Neurophysiology, Max Planck Institute for Brain ResearchFrankfurt, Germany; ^3^Frankfurt Institute for Advanced Studies, Johann Wolfgang Goethe UniversityFrankfurt, Germany; ^4^Communications Engineering Lab, Technische Universität DarmstadtDarmstadt, Germany; ^5^Department of Neuroinformatics, Institute of Cognitive Science, Universität OsnabrückOsnabrück, Germany

**Keywords:** parallel factor analysis, principal component analysis, cross correlation, cat primary visual cortex, cortical deactivation

## Abstract

The identification of important features in multi-electrode recordings requires the decomposition of data in order to disclose relevant features and to offer a clear graphical representation. This can be a demanding task. Parallel Factor Analysis (PARAFAC; Hitchcock, 1927; Carrol and Chang, 1970; Harshman, 1970) is a method to decompose multi-dimensional arrays in order to focus on the features of interest, and provides a distinct illustration of the results. We applied PARAFAC to analyse spatio-temporal patterns in the functional connectivity between neurons, as revealed in their spike trains recorded in cat primary visual cortex (area 18). During these recordings we reversibly deactivated feedback connections from higher visual areas in the pMS (posterior middle suprasylvian) cortex in order to study the impact of these top-down signals. Cross correlation was computed for every possible pair of the 16 electrodes in the electrode array. PARAFAC was then used to reveal the effects of time, stimulus, and deactivation condition on the correlation patterns. Our results show that PARAFAC is able to reliably extract changes in correlation strength for different experimental conditions and display the relevant features. Thus, PARAFAC proves to be well-suited for the use in the context of electrophysiological (action potential) recordings.

## 1. Introduction

Action potentials are the means of information transmission between neurons. Even though we have a broad knowledge about action potentials on the single-cell level (Bean, 2007), a thorough understanding of the principles of the neuronal code as a whole has not yet been achieved. In order to obtain a better insight into this challenging question, numerous different approaches have been used. Many of them take advantage of the possibility of simultaneous multi-electrode recordings (Buzsáki, 2004). Synchronous firing of action potentials is believed to be one of the crucial mechanisms of information coding in the brain (for reviews see Singer, 1999; Uhlhaas et al., 2009). Hence, the examination of the temporal structure of spike trains (sequences of action potentials) and the detection of correlations among the signals of multi-electrode recordings can provide fundamental insights into presumptive coding strategies. To this end, the temporal structure in parallel recordings of spike trains has been used to assess the flow of information among neurons (Grün et al., 2002; Pipa et al., 2008).

Different phenomena in the spiking activities of different neuron groups can be relevant for analysis. In the present study, we focus on two issues: First, the correlation of the spiking activities of different groups of neurons, which can be identified by a peak in the cross correlogram and is termed *synchrony*, second at periodically appearing correlation in the spiking activities, called *oscillatory synchrony*.

Since analysis of synchronous events becomes more demanding as soon as more than two event sources are considered, techniques that are able to identify synchrony among multiple simultaneously active sources are of great interest. In the present study, we use PARAllel FACtor analysis (PARAFAC) to assess the effects of deactivation of higher visual areas on patterns of spike synchronization in area 18 in cats (see Materials and Methods Section). PARAFAC is a multi-dimensional decomposition method that generalizes the bilinear principal component analysis (PCA) (Jolliffe, 2002) to higher order arrays. The method goes back to Hitchcock (Hitchcock, 1927) and was further developed simultaneously by Harshman Harshman (1970) and Carrol and Chang (Carroll and Chang, 1970), in the latter case called CANDECOMP (CANonical DECOMPosition). The same method has also been referred to as CPD (Canonical Polyadic Decomposition).

Here, we applied PARAFAC to three-dimensional arrays. A three-dimensional array can be simply viewed as a set of two-dimensional matrices of the same size. An example for a two-dimensional data array could be some measured variable, say the concentration of ozone (*O*_3_) in the air, at different times of the day in different geographical locations. Two-way arrays of this kind are often decomposed using (bilinear) Principal Component Analysis (PCA). Imagine you are now interested in the ozone concentration not only at certain times and geographical coordinates, but also at different altitudes. The measurements now become dependent on three variables, and your array three-dimensional. PCA cannot be applied to three-dimensional structures as it is inherently bilinear. If unfolded, the array can be subjected to PCA, but loses its true three-dimensional structure. PARAFAC is able to work directly on the three-dimensional array and thus capture its true composition. An alternative to PARAFAC can be provided by the Tucker3 algorithm, which is essentially a more flexible version of PARAFAC, meaning that any data set that can be modeled with PARAFAC can also be modeled with Tucker3 (Kiers, 1991; Bro, 1997). These methods can also be extended to n-way arrays with *n* > 3.

In general, there is not one best method, but one has to explore, which one is best for a given dataset. We chose PARAFAC as it was able to extract important information from our data and the approach could be validated by the amount of variance explained and a split-half analysis. PARAFAC is constrained to consider only full multiplicative interactions among the different dimensions, leading to simple mathematical models. The solution obtained by application of PARAFAC is unique, in contrast to PCA, where rotational freedom exists, which allows results to be rotated without reducing the quality of the modeling (Bro, 1997). Thus, PARAFAC enables robust multi-dimensional analyses that lead to a simple and clear interpretation. PARAFAC was first developed for application in psycho- and chemometric settings. In neuroscience, the method has been applied in EEG (Miwakeichi et al., 2004; Mørup et al., 2006) and fMRI (Beckmann and Smith, 2005) studies. However, it has so far not been tested on action potential recordings.

## 2. Materials and methods

### 2.1. Experimental methods

The analyzed dataset was collected in the course of a series of experiments investigating the role of feedback connections from the posterior middle suprasylvian (pMS) cortex to primary visual areas, in this case area 18 in the cat visual system. To this end, pMS cortex was thermally deactivated using cryoloops (Lomber et al., 1999), while spontaneous or visually evoked activity was recorded in area 18. This procedure is reversible and can therefore be applied numerous times without harming the deactivated brain area. There were four different deactivation conditions: (1) warm, i.e., no deactivation, (2) deactivation of pMS in the ipsilateral hemisphere with respect to the recording, (3) deactivation of pMS in the contralateral hemisphere, and (4) bilateral deactivation of pMS cortex. In the awake behaving animal it has been shown that unilateral deactivation of the pMS cortex results in visual hemineglect (Payne et al., 1996), similar to that described in human patients with a lesion in the parietal cortex (e.g., Rafal, 1994).

Interestingly, and also in accordance to findings in the human, this hemineglect in cats occurs only with unilateral lesions or deactivations of parietal cortex and is reversible once both parietal cortices are deactivated. Thus, this hemineglect phenomenon has to be seen as an attention deficit based on an interhemispheric imbalance rather than a form of blindness.

We applied the analysis methods described below to electrophysiological action potential recordings. For details on the surgical procedures and anesthesia see Galuske et al. (2002). Data were collected in an anesthetized male cat aged $1\frac{1}{2}$ years, using an Eckhorn multi-electrode matrix (Thomas Recording, Germany). Sixteen electrodes were placed in area 18 of cat visual cortex, with a distance of 500 μm in the x-y-plane, and individually moveable in the z-direction. Spike signals were recorded with a sampling rate of 22 kHz and band-pass filtered (800–5000 Hz). Multi-unit spike signals were obtained by thresholding. In addition, cooling loops (Lomber et al., 1999) had been placed over pMS cortex in order to induce a reversible deactivation of this region.

The animal was visually stimulated with a black and white high contrast square wave grating. The grating was presented in four different orientations (0°, 45°, 90°, 135°), which moved in either of the two directions perpendicular to the bars of the grating. Hence, eight different stimulus conditions were possible and the order of occurrence of the stimulus conditions was pseudo-randomized. Visual stimulation started with showing a gray screen for 2 s followed by one of the four differently oriented gratings remaining stationary for 2 s before the grating started moving for 4 s. Thus, one trial lasted for 8 s.

One cycle of the experiment consisted in showing 7 repetitions of each of the 8 stimuli in random order. The whole experiment consisted of 3 cycles baseline condition without deactivation, 3 cycles with thermal deactivation, followed by 3 cycles without deactivation after the affected regions of the brain had rewarmed.

All animal experiments were performed in accordance with the guidelines for the use of animals in research of the Society for Neuroscience and the local authorities and overseen by a local veterinarian (license number F122/08, Regierungspräsidium Darmstadt).

### 2.2. Synchrony and oscillatory synchrony

Two different metrics were established in order to quantify the degree of synchrony and oscillatory synchrony. Since both phenomena are identified using the correlogram, both metrics are based on the correlogram.

Spike trains were stored as binary vectors with sampling frequency resolution. Ones occurred where a sample value exceeded the chosen threshold and the sample before did not. For the calculation of cross correlations, a binning of 2 ms was introduced.

For the synchrony measure, the cross correlogram λ^raw^_*xy*_ of the spike trains **x** and **y** of two multi-units in the analysis window *T* was computed:

(1) $$\lambda_{xy}^{\text{raw}}\left(\tau \right):=\sum_{t\in T}x(t)y(t+\tau ).$$

To correct for rate induced chance coincidences, we normalized the correlograms to the firing rate. To this end, the spikes in the spike trains were convolved with a “jitter kernel” and the resulting cross correlogram was subtracted from the raw correlogram λ^raw^_*xy*_. To implement a computationally efficient jitter correction, we rely on convolution with a homogeneous filter with entries $\frac{T_{\text{sample}}}{T_{\text{jitter}}}$, with *T*_sample_ the time interval between two samples, which corresponds to jittering the spike train randomly within a time interval of *T*_jitter_ = 6 ms. The jittered spike train $\tilde{x}$ was thus obtained from spike train *x* according to $\tilde{x}$:= *K* * *x*, where * denotes the convolution. A correlogram was then computed from the two convolved spike trains and subtracted from the original correlogram, to obtain the normalized correlogram λ_*xy*_:= λ^raw^_*xy*_ − λ^jitter^_*xy*_. The convolution approach is equivalent to the random drawing of jitter times from a uniform distribution (following the Wiener-Khinchin theorem, see also Pipa et al., 2008).

The degree of synchrony was measured by the largest positive peak in the normalized correlogram within a window of size *T*_sync_ = 5 ms around a lag of zero. The synchrony metric κ_sync_ is thus defined as follows. Let $\kappa =\underset{l,l\in [-5\;\text{ms},5\;\text{ms}]}{\max}\lambda_{xy}(l)$. Then,

(2) $$\kappa_{\text{sync}}:=\{\begin{array}{ll} \kappa , & \text{ if }\kappa \geq 0\\ 0 & \text{ else}.\; \end{array}$$

In addition to the synchrony measure, we looked at the oscillatory synchrony between the spike trains. The degree of oscillatory synchrony can be determined by considering how much energy is contained in the oscillations of the correlogram in a chosen frequency range. To this end, the correlogram λ_*xy*_ was subjected to an N-point Discrete Fourier Transform (DFT) (Oppenheim et al., 1996), in order to extract the frequencies of interest.

The oscillatory synchrony κ_oscsync_ was then set as the relative power of the signal in a chosen frequency range between *f*_min_ and *f*_max_:

(3) $$\kappa_{\text{oscsync}}:=\frac{\sum_{m=f_{\text{min}}}^{f_{\text{max}}}DFT_{\lambda_{xy}}^{2}(m)}{\sum_{n=0}^{N-1}DFT_{\lambda_{xy}}^{2}(n)}.$$

#### 2.2.1. The PARAFAC model

PARAFAC is based on a mathematical model that represents the interactions of the dimensions in which the input data is to be analyzed. In order to carry out PARAFAC, the analysis dimensions have to be defined first. Each input value can then be related to an index for each of the dimensions. Assuming *N* = 3 dimensions, for example, *x_ijk_* identifies the measured value for index i in the first dimension, j in the second dimension and k in the third dimension. In our case, the three dimensions are constituted by three experimental variables: electrode pair, stimulus, and repetition/trial. The correlation values κ_sync_ are obtained by the procedure explained above and used as an input into the three-dimensional array. They are placed at the location corresponding to the experimental condition they were obtained for (electrode pair × stimulus × repetition/trial).

PARAFAC is now used to model this input array. Let F denote the number of so-called components and define so-called loading matrices A, B, and C of dimensions *I* × *F*, *J* × *F*, and *K* × *F* and with elements *a_if_*, *b_jf_*, and *c_kf_*, respectively, and the modeling error ε_*ijk*_. The general model used by PARAFAC to represent the input data is then given by (Bro, 1997)

(4) $$x_{ijk}=\sum_{f=1}^{F}a_{if}b_{jf}c_{kf}+\varepsilon_{ijk}.$$

**Figure 1:**
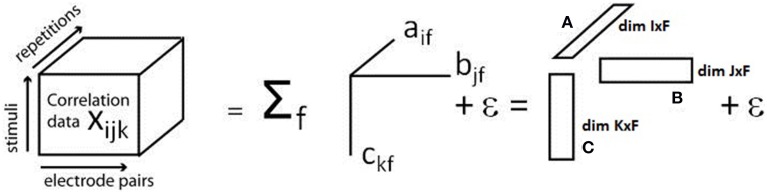
**Illustration of the PARAFAC model**. The three-dimensional array containing the cross correlation information for each electrode pair, stimulus, and repetition, is decomposed into a sum of products of three factors, called *loadings*, which build up the *loading matrices* A, B, and C. Modified with permission from Miwakeichi et al. (2004).

A graphical illustration of the model is given in Figure 1.

PARAFAC thus constrains the interactions between the different dimensions to the complete multiplicative interaction. The loading vectors are determined by minimizing the modeling error ε_*ijk*_. This minimization can be carried out using the alternating least squares (ALS) approach, for example. ALS iteratively determines the loading matrices A, B, and C by the following algorithm (Bro, 1997):

1. Choose the number of components, F (on the choice of F see next paragraph)
2. Initialize B and C
3. Estimate A from X, B, and C by least squares regression to minimize the square of the model error
4. Estimate B likewise
5. Estimate C likewise
6. Repeat from (3) until the algorithm converges (indicated by only little changes in fit or loadings)

The choice of the number *F* of components is difficult and no technique giving clear values has been identified yet. If *F* is chosen too small, not all effects in the input data can be identified. If *F* is chosen too large, however, noise is modeled increasingly and the existing effects in the data will be modeled by correlated components. Different approaches for estimation of the best value for *F* exist (Bro, 1997). The approach taken in this work was to increase the number *F* of components until the decrease in the residual error decayed significantly. The model with the optimal number of components was then determined to be the one which was able to explain the highest amount of variance without any of the components being correlated. The quality of the analysis results of multilinear variation strongly depends on the preprocessing of the input data. Possible preprocessing strategies are centering and scaling (see Bro, 1997): centering removes a non-zero mean from the data. A complete centering of the input data in all dimensions can be achieved by taking the result of the previous centering and center it in the next dimension. Scaling adjusts the variations in each of the dimensions to comparable magnitudes. Note that in contrast to centering, subsequent scaling of several modes is problematic since scaling one mode affects the scaling of other modes as well as the centering of the same mode. For this reason, centering should be carried out after scaling. Iterative approaches that can achieve a scaling of all modes are available (Bro, 1997). In this study, the data were centered in all dimensions. No scaling of the data was carried out.

### 2.3. Validation

The verification of the results obtained with the PARAFAC model of (5) was achieved by carrying out so-called split-half experiments (Harshman and Lundy, 1994). To this end, the set of input data is split into two halves and PARAFAC is carried out for both halves independently. The model is considered to be applicable if the results gained from both halves are similar. In this work, several split-half experiments were carried out, splitting the input data set into odd and even trials.

### 2.4. PCA

We use the trilinear PARAFAC model because we assume the data to be (at least) trilinear. To show that a bilinear model, such as principal component analysis (PCA), is not adequate in this context, we also decomposed the correlation matrices using PCA and compared the results. PCA is a widely used technique. An introduction can be found in Jolliffe (2002). In order to make the data array accessible for PCA, it was unfolded into a two-dimensional structure.

## 3. Results

**Figure 2:**
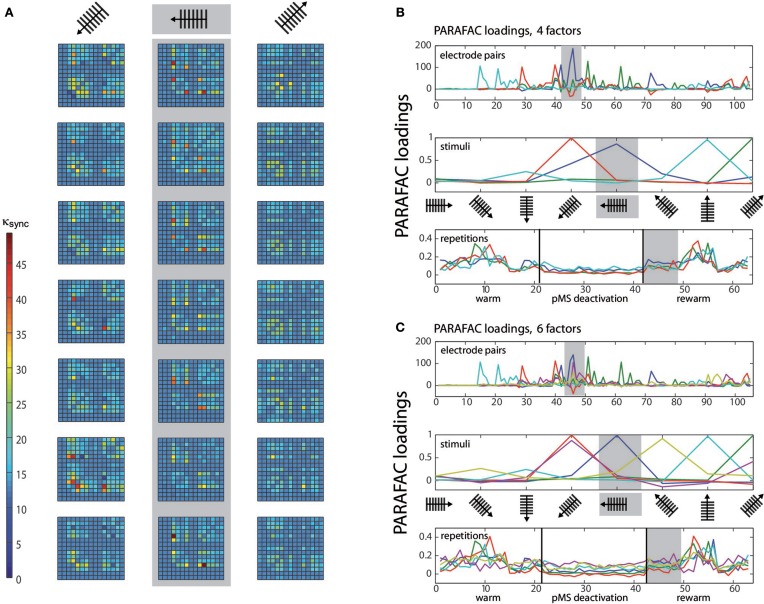
**PARAFAC visualization**. **(A)** Depicts the κ_*sync*_ measure for the three stimuli with the highest correlated activity. For each stimulus, seven repetitions are shown. κ_*sync*_, expressing correlation strength, is color-coded, see color bar. The correlations correspond to repetitions 43–49 (rewarm phase) in **(B)** and **(C)** (see gray shaded areas). The values κ_*sync*_ (electrode pair, stimulus, repetition) are subject to the PARAFAC analysis. **(B)** Shows the results of a 3D PARAFAC analysis for the choice of the dimensions electrode pairs, stimulus directions, and repetitions of the stimulus in a session with bilateral pMS deactivation. The algorithm was carried out with four factors. **(C)** shows the PARAFAC result for a calculation with six factors for the same dataset as in **(B)**. Note that for each factor, a new aspect of the correlation structure becomes visible, as contributions of new electrode pairs and stimuli emerge. The plots are to be interpreted in the following way: Values from the original array containing the values of κ_*sync*_ for the three conditions electrode pair, stimulus, and repetition are factorized into loadings for a number of factors that is chosen beforehand. To obtain the original values (but for a small error), the loadings for one factor are multiplied and then added over all factors. Loadings belonging to the same factor are marked in the same color. The dark blue factor in the third panel of **(B)** has the highest loading values for this phase. The electrode pairs and stimuli for which this finding holds are the ones whose dark blue factor also takes high loading values. This points to the fact that stimulus 5 (vertical grating, movement to the left) leads to the highest correlation values during this phase of the experiment, mostly for electrode pairs 45 and 72 (upper panel). The pairs emphasized by the PARAFAC loadings are the same ones that repeatedly show high correlation values in **(A)**, but their contribution is much more easily accessible using the PARAFAC visualization. To help with reading the plot, let us consider another example, concentrating on the light blue curve: As written in the top left corner of the plot, this panel depicts the influence of the electrode pair. Hence, correlation is high for e.g., electrode pair no. 15 (but also no. 21, for example). In order to find out if this is true for a specific stimulus, one should check the middle panel: here, it is shown that the effect is most prominent for the grating moving upwards. To get an idea of the time course of this effect, examine the lower panel of stimulus repetitions. This indicates that the high correlations were mainly observed in the middle of the warm and rewarm conditions (here, the loading values are high), e.g., especially for the 10 repetition. This procedure can be applied for the other factors as well, there is no order of importance.

To demonstrate the application of PARAFAC to multiunit spiking data, we applied the algorithm to an example dataset obtained in the experiment described above. PARAFAC was carried out in MATLAB (The Mathworks, Inc., Natick, MA) using the N-way toolbox developed by Andersson and Bro (Andersson and Bro, 2000, available at http://www.models.life.ku.dk/nwaytoolbox/). The customized program code is available as Supplementary Material on the article website. Figure 2A illustrates the synchrony measure, κ_*sync*_, for one experimental session (three stimulus directions are shown).

The first 21 repetitions of each stimulus were thus recorded without deactivation, repetitions 22–42 correspond to the phases of thermal deactivation of pMS and repetitions 43–63 show the results for the rewarm condition. In each of the subplots of Figure 2A, the 15 recording channels are shown on x-and y-axis (one channel in the 4 × 4 grid was not working and was not included in the analysis). Each square in the subplots represents the strength of correlation (κ_*sync*_) for the respective pair of recording channels. The figure provides an illustrative representation of the synchrony effects in the spiking data. It clearly shows that the signals of certain groups of neurons are correlated only for the presentation of certain stimuli. Furthermore, it can be observed that this structure is maintained over time. After examination of the correlation plots, we chose a 3D PARAFAC with the dimensions electrode pair, stimulus and repetition. The input data was centered in all dimensions. No scaling of the data was carried out. The measures based on normalized cross correlation data for all possible pairs of electrodes in the array were fed into the PARAFAC algorithm to obtain the multidimensional analysis results.

### 3.1. Synchrony

The PARAFAC algorithm revealed the influence of electrode pairs and stimuli on the correlations and showed its behavior over time, since the stimulus repetitions were considered in an extra dimension. Figure 2 shows the result of the PARAFAC analysis. The optimal number of factors was chosen as the highest number of factors with no correlated ones and resulted in numbers of 4–6.

The loadings can be interpreted as the strength of influence on the correlation for the respective electrode pair, stimulus condition, and time point: The higher the loading value, the stronger the influence of the respective feature on the correlation.

The correlation values in Figure 2A correspond to repetitions 43–49 in Figures 2B,C. A comparison of these corresponding parts shows that the same effects can be observed in both ways of presentation. This becomes especially clear when the focus is put on the dark blue PARAFAC component in Figures 2B for repetitions 43–49. The component clearly reflects the strong peaks in Figure 2A for the respective stimulus.

Separate analyses were carried out taking into account the distance between the electrodes. For this purpose, the electrodes in a homogenously spaced 4 × 4 grid with grid size *d_grid_* were divided into two sets, the set of neighboring pairs and the set of remote pairs. With (*x_i_*,*y_i_*) the position of electrode *i*, two electrodes are called neighboring nodes if

(5) $$\sqrt{{\left(x_{1}-x_{2}\right)}^{2}+{\left(y_{1}-y_{2}\right)}^{2}}\leq \sqrt{2}d_{grid}$$

holds. Any two electrodes fulfilling this constraint are referred to as neighboring pairs, all other electrode pairs are termed remote pairs. In the case of a 4 × 4 grid of electrodes, this results in 42 neighboring pairs and 78 remote pairs.

**Figure 3:**
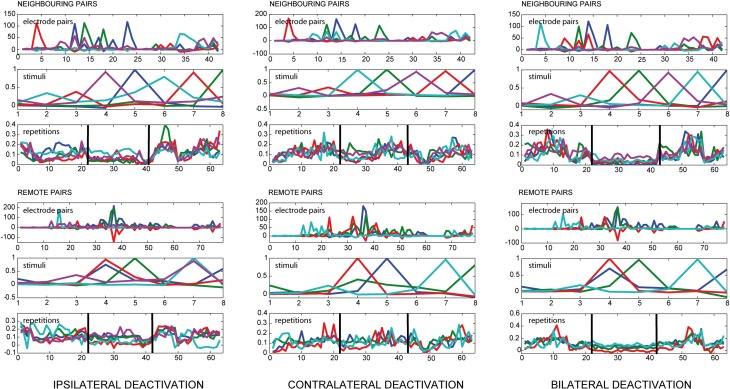
**Results of PARAFAC for local and remote connections**. The figure shows the results of a PARAFAC analysis for all deactivation conditions. The electrode pairs were split into neighboring and remote pairs according to the neighborhood condition defined in Equation (5). If one looks at the loading values for the repetitions, illustrating the time course of the observed correlations, the deactivation effect is visible for ipsilateral deactivation and even more pronounced during bilateral deactivation, depicted by a decrease in the PARAFAC loading value. During ipsi-and bilateral deactivation the variance for the PARAFAC loading values also decreases, indicating a network behavior which is less dynamic. During contralateral deactivation the course of the correlations does not seem to change substantially.

We found a strong influence of pMS deactivation on the strength of correlations for both ipsi- and bilateral deactivation (see Figure 3). For these conditions, loading values for the deactivation phase were considerably lower than for the warm phases. Also, the variation in the warm phases was higher, indicating a more dynamic correlation pattern. This effect could already be observed in the spontaneous activity before stimulus onset (results not shown).

During contralateral deactivation of pMS (see Figure 3), the effect was much weaker, showing that the activity in area 18 was almost not affected by the deactivation of contralateral pMS.

### 3.2. Oscillatory synchrony

**Figure 4:**
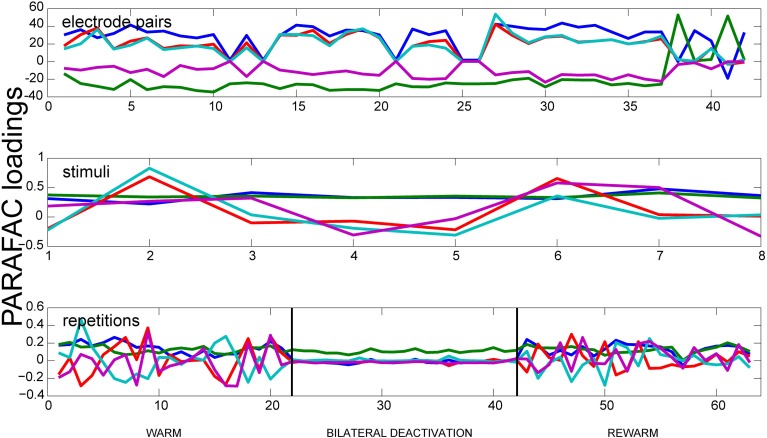
**Results of PARAFAC for the Oscillatory Synchrony measure**. Results of a PARAFAC analysis using the oscillatory synchrony measure is shown for a phase of spontaneous activity. Only neighboring electrode pairs are shown. The bilateral deactivation of the pMS cortex has a strong effect on the correlations, which can be clearly observed when looking at the deactivation phase in the lower panel.

In order to analyze the measured data with respect to its oscillatory synchrony properties, the oscillatory synchrony measure κ_*oscsync*_ was used as input data for the PARAFAC algorithm (Figure 4). For this measure the deactivation effects were most prominent during spontaneous activity. For frequencies between 1 and 10 Hz the deactivation effect could only be observed for bilateral deactivation. Here, it leads to a severe decrease of correlations. The correlations return to initial values in the rewarm phase. For the low gamma frequency range (30–50 Hz), the deactivation effect can also be seen during ipsilateral deactivation of pMS, and it is most pronounced during bilateral deactivation. In both frequency ranges, PARAFAC very soon developed correlated factors. For the oscillatory synchrony, the highest value for the variance explained by the model reached levels of 50% (for the results shown in Figure 4). This value increases when the number of factors F is increased. However, as already mentioned above, the factors become correlated under these conditions.

### 3.3. Validation

**Figure 5:**
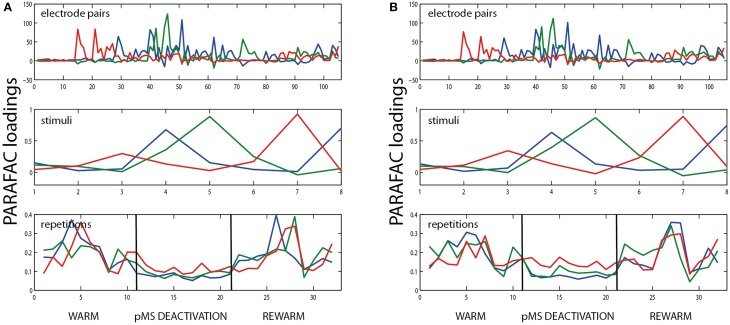
**Example for split half validation**. The figure shows the results of PARAFAC analysis of the bilateral deactivation data shown in Figure 2, split into two halves. **(A)** shows the results for all odd trial labels, **(B)** for the even-numbered trials. The similarity of the plots indicates that it is justifiable to use PARAFAC in this context.

As a control for the appropriateness of PARAFAC for our data, we performed split-half experiments in which the data set was divided into odd and even trials. Figure 5 shows an example of the results for the data shown in Figure 5. The results show that the method works equally well on both newly created data sets: The loadings are similar and also the variation explained by the model was similar in both cases (71.26 and 70.17%, respectively). Consequently, the PARAFAC model of (5) was considered to be applicable.

### 3.4. PCA

**Figure 6:**
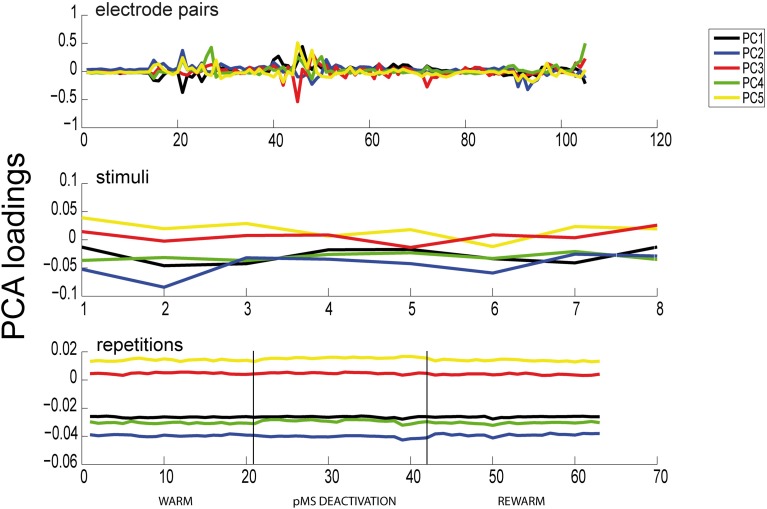
**Results of PCA**. Here we show the results of a PCA for the same dataset as previously analyzed with PARAFAC (see Figure 6). All 105 electrode pairs are shown. It can be observed that PCA is able to extract the same correlated pairs as PARAFAC, but the changes in correlations over time are not captured. Hence, the deactivation effect is not apparent in any of the panels.

The results of the PCA showed two distinct features: (1) The phases of pMS deactivation are not very prominent (see Figure 6) A high number of principal components is needed until a reasonable proportion of the total variance is explained. We showed that, for our example data set, with four and six PARAFAC components, the model is able to explain 70.07%, respectively 76.26% of the variance in the data. In comparison, 17 PCA components were needed to reach 70% of cumulated variance.

## 4. Discussion

Since PARAFAC applies a truly multilinear model (Bro, 1997), it is a well-suited decomposition technique for data that can be factorized. In our experiments, stimulus condition and repetition can be assumed to be independent, such that they can be factorized. Thus, PARAFAC can be expected to be suitable for data analysis in the scope of our study. The results of the analysis of correlation effects in action potential data, as shown in Figures 2B,C, support this reasoning. They show that PARAFAC allows to easily assess the correlation effects in the data and to represent the relevant dependencies much more compact than more elementary analysis techniques, such as the evaluation of plain spike rates or correlations as shown in Figure 2A. Thus, PARAFAC proves to be a good choice to reveal the structure contained in the experimental data and to enable the assessment of the functional relation between the recorded groups of neurons as well as the influence of different stimulus conditions and time structure.

In contrast to PARAFAC, PCA is not a multilinear but a bilinear decomposition (Smilde and Doornbos, 1991). If applied to multi-dimensional data, PCA requires unfolding of the multidimensional data array to a two-dimensional matrix. Unfolding easily causes overfitting since the two-dimensional structure of the model does not correspond to the underlying structure of the data. Furthermore, PARAFAC applies a less complex model (as compared to PCA), which features easier and clearer interpretation. In particular, the uniqueness of the solutions of PARAFAC has to be emphasized in this context, since this is in strong contrast to the solutions obtained with PCA, which provide rotation invariance (Bro, 1997). The choice of PARAFAC as the decomposition technique is also supported by the principle of parsimony (Seasholtz and Kowalski, 1993; also “Occams razor”), which recommends to use the least complex model that is capable of capturing the desired information.

Concerning the determination of the solutions, more effort has to be spent when using PARAFAC instead of PCA. This is mainly due to two reasons. First, the determination of the number of components required for the PARAFAC is not trivial and requires effort prior to the actual determination of the solution. Second, obtaining the solution of the PARAFAC using the alternating least squares (ALS) algorithm is computationally more complex than the projection applied in PCA. In addition, it has to be kept in mind that with PARAFAC, the solutions obtained for different number of components are independent, such that changing the number of components requires the recalculation of the complete solution.

One prerequisite for the use of the ALS algorithm is a normal distribution of the model error ε_*ijk*_. However, this could not be held for our dataset, therefore the validity of our analysis has to be tested. To this end, split-half experiments were carried out. The results are shown in Figure 2. The results show that despite an error distribution that deviates from the normal distribution, the PARAFAC results obtained with the ALS algorithm are stable and meaningful. For further analysis of the sensitivity of the PARAFAC solution with respect to the error distribution, a bootstrapping procedure (Efron and Tibshirani, 1993) that works with two different sets of errors is suggested: One set is obtained by randomly drawing error values from the empirical distribution of errors as they actually occurred in the PARAFAC analysis. The second set of errors is obtained from a Gaussian distribution that is fitted to the empirical distribution of the empirical errors. The errors from both sets are added to the original data and a PARAFAC analysis is carried out for the two data sets. The sensitivity of the PARAFAC algorithm to the distribution of the error can then be determined by comparing the solutions of PARAFAC obtained from the two data sets.

To summarize, PARAFAC is proposed in this work as a technique to analyze action potential recordings. The proposal is made based on the properties of PARAFAC and the properties of the data. The application of PARAFAC for the proposed purpose is verified in this work and the analysis results support the reasoning. A comparison with other relevant approaches is drawn and the differences between PARAFAC and other approaches are discussed. Furthermore, the specific requirement of PARAFAC concerning the normal distribution of the analysis error is discussed and the effect of an error distribution that deviates from the normal distribution on the analysis performance is evaluated. It is shown that the proposed analysis approach is valid also for a non-normal distribution of the analysis error. For a more detailed analysis of the sensitivity of the analysis, a bootstrapping approach is proposed.

### 4.1. Discussion of results

Even though this is a very methodological contribution we would like to illustrate how far this unique combination of different methodological approaches may help to reveal the neuronal basis of brain states. It should be emphasized that we only analyzed a randomly selected set of data from these experiments in order to examine to what extent the application of PARAFAC could help to better describe brain states and their transition. Unilateral deactivation of pMS cortex is linked to the neurological syndrome of visual neglect, an attentional deficit with impairment in motion, spatial, and attentional processing in one half of the visual field (Lomber, 2001). Bilateral pMS deactivation, by contrast, leads to a remarkable restitution of behavior (Lomber and Payne, 1996). Our PARAFAC analysis is able to reveal distinct effects of the deactivation of the pMS cortex on correlated activity in area 18 (see Figure 3, left and right columns, middle parts of bottom panels): during ipsi-, and even more during bilateral pMS deactivation, the strength of correlated activity in area 18 decreases. This is in accordance with our expectations, as unilateral deactivation of the pMS cortex eliminates the strong feedback input from pMS to the ipsilateral area 18 (Symonds and Rosenquist, 1984). However, while the feedback input from the ipsilateral pMS is missing, the affected primary visual area still gets feedforward input from LGN and also lateral input from its non-affected contralateral homotopic area and the contralateral pMS (Segraves and Innocenti, 1985).

When the middle panels of Figures 2B,C are examined, the analysis seems to allocate the factors in such a way that each factor depicts the situation for one distinct stimulus condition. For each of the stimuli, a certain pattern of correlated electrode pairs emerges, i.e., a different network is observed. In Figure 2C, with the 6th factor (shown in magenta), another set of correlated electrode pairs that is present when a diagonally left/downwards moving stimulus is presented, becomes visible. The same electrode pairs are also correlated for the stimulus moving in the opposite direction, and a slight anticorrelation can be observed for the perpendicular stimulus. The bottom panels show the time course of effects, indicating a larger trial-to-trial variability during the warm phases as compared to the ipsi- and bilateral cooling sequences and similar variability for the warm condition and contralateral cooling (see Figure 3). Hence, each stimulus produces a distinct network activity, but all of those networks become less variable during the ipsi- and bilateral deactivation phases.

The neuronal correlate of visual neglect has been proposed to be based on an imbalance of activity between hemispheres, which, presumably among other effects, leads to a disinhibition of areas in the non-affected hemisphere, which in turn leads to further inhibition of the already attenuated area (Payne and Rushmore, 2004). This is in accordance with the low level of correlated activity in ipsilateral area 18 (relative to the deactivation) and the observed correlation level in the contralateral area 18, which resembles the warm condition. When both pMS cortices are deactivated, as in the bilateral deactivation condition, the input from the contralateral areas is also reduced. Hence, the degree of correlated activity in area 18 is further lowered. In addition to the strength of correlation, the trial-to-trial variability is also diminished (see Figure 3, right column). This could indicate a less dynamic neuronal network, operating on a lower activity level, which is less able to adapt to and process new stimulus inputs than the normally functioning visual network. However, the lower but balanced correlation level seems to enable at least some functions that are impaired by unilateral deactivation: Behavioral studies indicate that during bilateral deactivation of pMS cortex, the animal is still able to complete simple orienting tasks, while it encounters problems with more abstract tasks, such as the so-called landmark-discrimination task, in which the cat is asked to choose between two wells, one of which contains food, the correct side of the well-being cued by one of six equally spaced landmarks, three left and three right of the midline (Lomber and Payne, 2000). Thus, although the animal is able to successfully complete some tasks, which may be owed to the restored equilibrium of activity levels in both hemispheres, there is an impairment of cognitive functions. Figure 4 shows that a strong decrease in the variation of correlated activity is even present in the spontaneous activity before stimulus onset, indicating a different processing mode of the respective neuronal network (see bottom panel, deactivation phase). As these are only speculations based on a very preliminary analysis of only a part of the dataset, these findings and conclusions have to be validated on the full database from these experiments. Also, further analyses should be conducted to examine the detected effects in greater detail. Nevertheless, PARAFAC proved to be a highly sensitive and helpful tool to identify relevant changes in neuronal processing modes on a multi-dimensional scale which open the window for a more targeted analysis of brain states linked to perceptual (and other) performance.

## Funding

This work was partially supported by the EU-project PHOCUS (FET-Open 240763) (Gordon Pipa, S. Katharina Schmitz).

### Conflict of interest statement

The authors declare that the research was conducted in the absence of any commercial or financial relationships that could be construed as a potential conflict of interest.
